# Diagnostic accuracy of the syndromic management of four STIs among individuals seeking treatment at a health centre in Nairobi, Kenya: a cross-sectional study

**DOI:** 10.11604/pamj.2021.40.138.25166

**Published:** 2021-11-04

**Authors:** Anne Njeri Maina, Marianne Wanjiru Mureithi, John Kiiru Ndemi, Gunturu Revathi

**Affiliations:** 1 Department of Medical Microbiology, University of Nairobi, Nairobi, Kenya,; 2 Kenya Medical Research Institute, Nairobi, Kenya,; 3 Aga Khan University Hospital, Nairobi, Kenya

**Keywords:** Sexually transmitted diseases (STIs), syndromic, diagnostic accuracy, Kenya

## Abstract

**Introduction:**

the syndromic approach to the management of sexually transmitted diseases (STIs) is recommended in areas without adequate laboratory support. We assessed the diagnostic accuracy of this approach in diagnosing Neisseria gonorrhoeae (NG), Chlamydia trachomatis (CT), Trichomonas vaginalis (TV) and Mycoplasma genitalium (MG) among 18 to 49 year-old individuals seeking treatment for STIs in a health centre in Nairobi, Kenya.

**Methods:**

participants were recruited between April and June 2019. After providing written informed consent, an interviewer-administered questionnaire was completed. Endocervical swabs from women and urethral swabs from men were collected for STI testing using polymerase chain reaction (PCR). Diagnostic accuracy of reported symptoms was calculated using PCR as the gold standard.

**Results:**

a total of 297 individuals (148 men and 149 women) were recruited. Majority of the participants had at least one reported symptom (130/148; 87.8% men and 145/148; 97.3% women). The most commonly diagnosed STI was NG (85/297; 28.6% 95%CI 23.5%-34.1%). Vaginal discharge syndrome had moderate (44.4%) to high (92.9%) sensitivity, low specificity, low positive predictive value (PPV) (2.4 % to 31.5%) and high negative predictive value (NPV) (68.2% to 95.2%). The lower abdominal pain syndrome had moderate to high sensitivity (40% to 71.4%), low specificity (30.9% to 35.6%), low PPV (9.9% to 15.8%) and high NPV (79.2% to 93.8%). The urethral discharge syndrome had high sensitivity (71.4% to 84.8%); moderate specificity (37.6% to 51.7%); low to moderate PPV (5.4% to 53.8%) and high NPV (83.6% to 96.4%). The kappa scores for the three syndromes were generally poor.

**Conclusion:**

these findings support the need for the review of the syndromic management of STIs.

## Introduction

The syndromic management of sexually transmitted infections (STIs), described by the World Health Organisation (WHO), is recommended in settings lacking adequate laboratory support [1]. Kenya adopted this approach in the mid 1990s which has remained the mainstay of STI management [2]. It involves the use of an algorithm of commonly presenting symptoms to identify commonly occurring infections and subsequent use of antimicrobial agents to treat organisms associated with these infections. Most of the time, a cocktail of antimicrobials is advocated as a package to cover multiple pathogens, leading to excessive and unnecessary use of antimicrobials promoting massive resistance among pathogens as well as normal uro-genital flora [1].

While adequate diagnostic accuracy has previously been demonstrated for the syndromic approach in men, it has consistently demonstrated poor performance in women [3]. This poor performance in women is especially evident in screening for cervical infections and the fact that cervical infections can be asymptomatic [4,5] further complicates its use. As a result, asymptomatic women remain untreated and at risk of sequelae such as pelvic inflammatory disease (PID), tubal factor infertility and poor pregnancy outcomes [6] among others.

Poor diagnostic accuracy also leads to overtreatment [3]. This is especially worrisome with increasing reports of antimicrobial resistance especially by *Neisseria gonorrhoeae* (NG) and *Mycoplasma genitalium* (MG) [7], and the evidence that exposure of microorganisms to antimicrobial agents is associated with development of resistance [8]. To counter these, the aetiologic approach to management of STIs is preferred over the syndromic approach. Indeed, in its Global Health Sector Strategy on STIs 2016-2021, the WHO aims to improve access to point-of-care tests (POCTs) as a way to move from syndromic management to aetiologic management of STIs [9]. For this to happen, countries need to understand their STI data and especially the aetiology of the common syndromes.

The Kenyan syndromic management guidelines were revised in 2018 [2]. The WHO recommends an aetiologic assessment for countries using syndromic management every few years [9]. We therefore sought to investigate the diagnostic accuracy of the urethral discharge syndrome (UDS), the vaginal discharge syndrome (VDS) and the lower abdominal pain syndrome (LAPS) in the diagnosis of *Neisseria gonorrhoeae, Chlamydia trachomatis* (CT), *Trichomonas vaginalis* (TV) and *Mycoplasma genitalium*.

## Methods

We enrolled individuals, 18 to 49 years, seeking treatment for STIs at the Special Treatment Centre (STC) Casino Health Centre in Nairobi, Kenya. The health centre serves members of the general population from Nairobi County and its environs and is also a well-known centre for the treatment of STIs.

**Sample size calculation:** this was a baseline survey for a case-control study on the immune response to *Neisseria gonorrhoeae* infection. Sample size for the main study was estimated using the formula described by Dupont and Plummer for the comparison of means for two groups [10];


$$n=\left(\frac{r+1}{r}\right)\frac{\sigma^{2}{\left(Z_{\beta }+Z_{\alpha /2}\right)}^{2}}{{\left(\mu_{cases}-\mu_{controls}\right)}^{2}}$$

The formula gives the minimum number of case subjects required to detect a true mean difference with power (1 - β) and two-sided type I error probability α (alpha). Where: n is number of exposed subjects; Z_α/2_ is the standard normal variate for level of significance (typically 1.96 for 95% confidence level); Z_β_ is the standard normal variate for power (typically 0.84 for 80% power); r is the ratio of non-exposed subjects to exposed subject=1; α is the standard deviation of the outcome variable estimated from previous studies =10 [11]; (µ_cases_- µ_controls_) is the effect size = 5. Substituting:


$$n=\left(\frac{1+1}{1}\right)\frac{10^{2}{\left(0.84+1.96\right)}^{2}}{{\left(5\right)}^{2}}=63$$

Total sample size for two groups was 126 (63 NG positive cases (CT, MG and TV negative) and 63 NG negative controls (NG, CT, TV and MG negative)). Convenient sampling was therefore done until the required sample size for the main study was obtained.

**Participant enrolment:** individuals were informed about the study and invited to participate. A written informed consent was sought from those willing to participate and who met the inclusion criteria. Pregnant women, menstruating women and those who had used either oral or intravaginal antimicrobial agents two weeks prior to recruitment were excluded.

**Data collection:** an interviewer-administered questionnaire was used to capture socio-demographic characteristics, symptoms and risks for acquisition of STIs, contraception and menstrual history for women and circumcision status for men, among others. Using Kenya´s guidelines, we collected data on reported symptoms for UDS from men; VDS and LAPS (LAP, vaginal discharge, dysuria, and abnormal bleeding (post-coital bleeding and intermenstrual bleeding)) from women.

Endocervical swabs from women and urethral swabs from men (Puritan®, Puritan Medical Products, Maine USA) were taken and placed on ice immediately after collection. Transportation was done within 2 hours to the Department of Medical Microbiology, University of Nairobi, where they were stored at -70° C until processing.

All participants were tested for HIV using Determine HIV Rapid Test (Abbott Diagnostics) with confirmatory testing being done with the First Response™ (Premier Medical Corporation Private Ltd., Gujarat, India), according to Kenya´s Ministry of Heath guidelines. Those who tested negative with the first test were considered HIV negative while those who tested positive with the confirmatory test were considered HIV positive and were either enrolled in the Casino H/C HIV Comprehensive Care Clinic (CCC) or referred to a health facility of their choice for follow-up. Screening for *Neisseria gonorrhoeae, Chlamydia trachomatis, Trichomonas vaginalis* and *Mycoplasma genitalium* was done using Multiplex PCR (Sacace Biotechnologies, Como, Italy) according to the manufacturer´s instructions. Participants received a KShs. 500 (~5 USD) reimbursement to compensate for the extra time spent at the clinic during the study procedures.

**Data handling and analysis:** data were entered and managed using REDCap electronic data capture tools hosted at APHRC [12]. Analysis was done using STATA version 15.1. Ethical approval was provided by the Kenyatta National Hospital/University of Nairobi ethical review committee (KNH-UoN ERC), protocol number (P304/06/2017). Permission to conduct the study at casino H/C was granted by Nairobi County´s Department of Health Services. After the laboratory results, participants were contacted by telephone and given an appointment to relay results. In the cases where participants had already been treated using the syndromic management guidelines, treatment was not repeated if they presented no additional complaints. They were also encouraged to disclose the diagnosis to their sexual partners.

Descriptive statistics were used to characterize sociodemographic variables disaggregated by gender. Pearson χ^2^test was used to assess the association of having any STI with various sociodemographic characteristics. Diagnostic accuracy (sensitivity, specificity, positive predictive value (PPV), negative predictive value (NPV)) was measured using laboratory-confirmed infections as the gold standard. We calculated Cohen´s kappa coefficient [13] to evaluate the degree of agreement between the gold standard and the reported symptoms. We used the interpretation of kappa values for levels of agreement described by McHugh (2012) [14] viz: <0 (agreement worse than expected); 0-0.20 (none); 0.21-0.39 (minimal agreement); 0.40-0.59 (weak agreement); 0.60-0.79 (moderate agreement); 0.80-0.90 (strong agreement); >0.90 (almost perfect agreement).

## Results

Between April and June 2019, we consecutively recruited 297 participants. Of these, 148/297 (49.8%) were male and 149/297 (50.2%) were female. Mean age of the participants was 30.8 years with majority being between 25 and 29 years (21.9%). Most participants were married (54.6%), had a secondary level education (39.5%), were self-employed (33.6%) and had an average monthly income of less than KShs.10,000 (~100USD) (48.0%) (Table 1). Contraceptive use was reported by 80/149 (53.7%) female participants with the majority using oral pills (30.0%). Most male participants were circumcised (93.2%). The mean age of sexual debut was 18.8 years with more participants having had sexual debut after 18 years of age (189/268; 70.5%). Overall, most participants reported having had one sexual partner (141/297; 47.5%) in the preceding year. Of those who reported a new sexual partner in the preceding three months, only 21.6 % reported condom use (Table 1 (suite)).

**Table 1:** sociodemographic and reproductive health characteristics and association with any STI

	Male (n=148)		Female (n=149)		All (n=297)		P value
**Characteristic**	**n**	**%**	**n**	**%**	**n**	**%**	
Age (mean, SD)	32.0	8.0	29.7	7.6	30.8	7.9	0.191
**Age categories**							**0.728**
18-19	4	2.7	10	6.7	14	4.7	
20-24	24	16.2	37	24.8	61	20.5	
25-29	32	21.6	33	22.2	65	21.9	
30-34	33	22.3	28	18.8	61	20.5	
35-39	21	14.2	19	12.8	40	13.5	
40-44	20	13.5	16	10.7	36	12.1	
45-49	14	9.5	6	4.0	20	5.7	
**Marital status**							**0.250**
Single	52	35.1	65	43.6	117	39.4	
Married	85	57.4	77	51.7	162	54.6	
Widow/widower	2	1.4	2	1.34	4	1.4	
Separated/divorced	9	6.1	5	3.4	14	4.8	
**Education level (n=296)**							**0.051**
None	7	4.7	2	1.4	9	3.0	
Primary	40	27.0	32	21.6	72	24.3	
Secondary	56	37.9	61	41.2	117	39.6	
College/university	45	30.4	53	35.9	98	33.1	
**Occupation**							**M:0.066; F:0.774**
Student	12	8.11	16	10.8	28	9.4	
Housewife			7	4.7	7	2.4	
Casual labourer	29	19.6	6	4.0	35	11.8	
Self-employed	54	36.5	50	33.6	104	35.0	
Salaried	49	33.1	46	30.9	95	32.0	
Unemployed	4	2.7	24	16.1	28	9.4	
**Monthly income (KShs.)**							**0.610**
<10,000	57	38.5	85	57.4	142	48.0	
10,001-20,000	57	38.5	44	29.7	101	34.1	
20,001-30,000	22	14.9	14	9.5	36	12.2	
30,001-40,000	2	1.3	2	1.3	4	1.4	
>40,000	10	6.8	3	2.0	13	4.4	

SD: standard deviation; *any STI (NG, CT, TV or MG infection)

**Table 1(suite):** sociodemographic and reproductive health characteristics and association with any STI*

	Male (n=148)		Female (n=149)		All (n=297)		P value
**Sexual debut (mean, SD) n=268**	**17.9**	**(3.7)**	**19**	**(3.3)**	**18.8**	**(3.6)**	**0.117**
**Sexual debut by age category**							**0.016**
<18 years	51	38.6	28	20.6	79	29.5	
≥ 18 years	81	61.4	108	79.4	189	70.5	
**Sexual orientation**							
Heterosexual	148	0	146	98.0	294	99.0	
Homosexual	0	0	1	0.7	1	0.3	
Bisexual	0	0	2	1.3	2	0.7	
**Type of contraceptive† (n=80)**							**0.634**
Condoms			20	25.0			
Injectables			7	8.8			
Oral pills			24	30.0			
IUCD			11	13.8			
Implant			9	11.3			
BTL			4	5			
Other			5	6.3			
**Circumcision**							**0.358**
Yes	138	93.2					
No	10	6.8					
**Sexual partners in the last one year**							**<0.0001**
1	42	28.4	99	66.4	141	47.5	
2	52	35.1	34	22.8	86	29.0	
3	24	16.2	10	6.7	34	11.5	
4	6	4.1	0	0	6	2.0	
>4	24	16.2	6	4.0	30	10.1	
**New sexual partner in the last 3 months**							**<0.0001**
No	75	50.7	120	80.5	195	65.7	
Yes	73	49.3	29	19.5	102	34.3	
**Condom use with new sexual partner (n=102)**							**0.235**
No	58	79.5	22	75.9	80	78.4	
Yes	15	20.6	7	24.1	22	21.6	
**Reported treated STI in last 3 months (n=296)**							**0.611**
No	116	78.4	127	85.8	243	82.0	
Yes	32	21.6	21	14.2	53	18.0	

SD: standard deviation; †female respondents only; *any STI (NG, CT, TV or MG infection)

**Laboratory diagnosed STIs:** overall, 147/297 (49.5%; 95%CI 43.7%-55.3%) participants were diagnosed with at least one of the four STIs. The most commonly diagnosed STI was NG (85/297; 28.6%; 95%CI 23.5%-34.1%) both in men (59/148; 39.9%; 95%CI 31.9%-48.2%) and women (26/149; 17.4%; 95%CI 11.7%-24.5%). Computed tomography (CT) was diagnosed in 50/297 individuals (16.8%; 95%CI 23.5%-34.1%; men: 32/148: 21.6%; 95%CI 15.3%-29.1%; women: 18/149; 12.1%; 95%CI 7.3%-18.4% ). More women (14/149; 9.4% 95%CI 5.2%-15.3%) than men (7/148; 4.7% 95%CI 1.9%-9.5%) were diagnosed with TV. In contrast, more men (12/148; 8.1%; 95%CI 4.3%-13.7%) than women (9/149; 6.0%; 95%CI 2.8%-11.2%) were diagnosed with MG. The commonest concurrent infection was NG/CT 15/297 (5.1%; 95%CI 2.9%-8.2%). HIV was diagnosed in 9/297(3.0%; 95%CI 1.4%-5.7%; men: 3.4 % 95%CI 1.1%-7.7%; women: 2.7 % 95%CI 0.7%-6.7%) individuals.

The number of sexual partners in the previous one year, having a sexual debut age below 18 years and having a new sexual partner in the previous 3 months had a statistically significant association with having any STI (Table 1 (suite)).

To further assess the association of having STIs with age, we classified participants into two age groups: <25 years and 25 years and above. The commonest infections for participants <25 years were CT in women (23.4%; 95%CI 12.3-38.0%) and NG in men (50.0%; 95% CI 30.6%-69.4%). NG was the commonest infection in both men and women over the age of 25 (37.5%; 95%CI 28.8%-46.8% and 18.6 %; 95%CI 11.6%-27.6% respectively). CT infection was associated with being less than 25 years of age in women (p=0.004) but not in men (p=0.321) (Table 2). Majority of the participants had at least one reported symptom (130/148; 87.8% men and 145/148; 97.3% women) with the most common being dysuria in men (122/148; 82.4%) and discharge in women (127/149; 85.2%). All MG infections in men and all TV infections in women were symptomatic. However, 14.3% of men with TV and 22.2% of women with MG had no reported symptoms.

**Table 2:** STIs by age category

	< 25 years		≥ 25 years		P value
**Male Participants**	**n=28**		**n=120**		
	**n**	**% (95% CI)**	**n**	**% (95%CI)**	
HIV (n=5)	0	0 (0.0-0.2)	5	4.2(1.4-9.5)	0.272
Any STI* (n=94)	19	67.9 (47.6-84.1)	75	62.5(53.2-71.2)	0.596
NG (n=59)	14	50.0 (30.6-69.4)	45	37.5(28.8-46.8)	0.224
CT (n=32)	8	28.6 (13.2-48.7)	24	20.0(13.3-28.3)	0.321
TV (n=7)	2	7.1(0.9-23.5)	5	4.2(1.4-9.5)	0.504
MG (n=12)	2	7.1(0.9-23.5)	10	8.3(4.1-14.8)	0.835
NG/CT (n=10)	4	14.3(4.0-32.7)	6	5.0(1.9-10.6)	0.078
**Female participants**	**n=47**		**n=102**		
HIV (n=4)	1	2.1 (0.0-11.3)	3	2.9(0.6-8.4)	0.775
Any STI* (n=53)	18	38.3(24.5-53.6)	35	34.3(25.2-44.4)	0.637
NG (n=26)	7	14.9(6.2-28.3)	19	18.6(11.6-27.6)	0.577
CT (n=18)	11	23.4(12.3-38.0)	7	6.9(2.8-13.6)	0.004
TV (n=14)	2	4.3(0.5-14.5)	12	11.8(6.2-19.6)	0.144
MG (n=9)	4	8.5(2.4-20.4)	5	4.9(1.6-11.1)	0.390
NG/CT (n=5)	3	6.4(1.3-17.5)	2	2.0(0.2-6.9)	0.164

### Diagnostic accuracy

**Urethral discharge syndrome:** in men, reported discharge alone had high sensitivity (71.4% to 84.8%); moderate specificity (37.6% to 51.7%); low to moderate PPV (5.4% to 53.8%) and high NPV (83.6% to 96.4%). Using reported discharge alone would have led to missed treatment rates of between 15.2% for NG and 28.6% for TV and overtreatment rates of between 48.3% for NG to 62.4 % for TV. The level of agreement between the reported discharge and the gold standard ranged from minimal (0.33; NG) to none (0.01; TV) (Table 3).

**Table 3:** performance of reported symptoms

	Sens	Spec	PPV	NPV	Missed treatment	Over treatment	Correct treatment	k
**Male participants**								
**Urethral discharge syndrome**								
**Dysuria**								
NG	89.8	22.5	43.4	76.9	10.1	77.5	49.3	0.10
CT	84.4	18.1	22.1	80.8	15.6	81.9	32.4	0.01
TV	85.7	17.7	4.9	96.2	14.3	82.3	21.0	0.00
MG	91.7	18.4	9.0	96.2	8.3	81.6	24.3	0.02
NG/CT	80.0	17.4	6.6	92.3	20.0	82.6	21.6	-0.004
**Discharge**								
NG	84.8	51.7	53.8	83.6	15.2	48.3	64.9	0.33
CT	75.0	40.5	25.8	85.5	25.0	59.5	48.0	0.10
TV	71.4	37.6	5.4	96.4	28.6	62.4	39.2	0.01
MG	83.3	39.0	10.8	96.4	16.7	61.0	42.6	0.05
NG/CT	80.0	38.4	8.6	96.4	20.0	61.6	41.2	0.04
**Dysuria and discharge**								
NG	81.4	58.4	56.5	82.5	18.7	41.6	67.6	0.37
CT	68.8	45.7	25.9	84.1	31.2	61.0	50.7	0.10
TV	71.4	43.3	5.9	96.8	28.6	56.7	44.6	0.02
MG	75.0	44.1	10.6	95.2	25.0	55.9	46.6	0.05
NG/CT	80.0	44.2	9.41	96.8	20.0	55.8	46.6	0.05
**Female participants**								
**Vaginal discharge syndrome (vaginal discharge or pruritus)**								
**Vaginal discharge**								
NG	73.1	12.2	15.0	68.2	26.9	87.8	22.8	-0.06
CT	72.2	13.0	10.4	77.3	27.8	87.0	20.1	-0.04
TV	92.9	15.6	10.2	95.5	7.1	84.4	22.8	0.02
MG	44.4	12.1	3.2	77.3	55.6	87.9	14.1	-0.06
NG/CT	60.0	13.9	2.4	90.9	40.0	86.1	15.4	-0.02
**Pruritus**								
NG	53.9	41.5	16.3	81.0	46.1	58.5	43.6	-0.02
CT	61.1	42.8	12.8	88.9	38.9	57.2	45.0	0.01
TV	71.4	43.7	11.6	93.7	28.6	56.3	46.3	0.05
MG	33.3	40.7	3.5	90.5	66.7	59.3	40.3	-0.05
NG/CT	40.0	41.7	2.3	95.2	60.0	58.3	41.6	-0.02

Sens: sensitivity; spec: specificity; PPV: positive predictive value; NPV: negative predictive value; LAP: lower abdominal pain; *abnormal bleeding = postcoital bleeding and intermenstrual bleeding

Similarly, reported dysuria had high sensitivity (80.0% to 91.7%); low specificity (17.4% to 22.5%); low to moderate PPV (6.6% to 43.4%); and high NPV (76.9% to 96.2%). Diagnosis based on reported dysuria alone would have resulted in between 8.3% of men with MG to 28.6% with TV missing treatment. Overtreatment rates would have been high (77.5% for NG to 82.6% for NG/CT) while correct treatment rates would have ranged from a low of 21.0% for TV to a high of 49.3% for NG. The kappa scores ranged from -0.004 for NG/CT to 0.10 for NG (Table 3). Using both symptoms would have led to a decrease in overtreatment rates and an improvement in correct treatment rates, except for CT. Further, the kappa scores improved for NG, TV and NG/CT (Table 3).

**Vaginal discharge syndrome:** in women, sensitivity of vaginal discharge ranged from moderate (44.4% for MG) to high (92.9% for TV); specificity was low. PPV was low (2.4 % to 31.5%) as were rates of correct treatment (14.1% to 32.9%). The rates of overtreatment were high for the use of discharge for diagnosis of all STIs. The rates of missed treatment were highest with NG/CT (60%) and lowest for TV (28.6%). The kappa score was poor (Table 3). Pruritus had moderate to high sensitivity, moderate specificity, low PPV and high NPV. Use of reported pruritus alone would have resulted in less than half of women with the four STIs getting the correct treatment. The kappa score was poor. Combining the two symptoms reduced rates of overtreatment and improved the rates of correct treatment for all the STIs compared to the use of individual symptoms. However, the PPV and NPV were generally low and the kappa scores were poor (Table 3).

**Lower abdominal pain syndrome:** use of reported LAP alone had moderate to high sensitivity (40% for NG/CT to 71.4% for TV) and low specificity (30.9% to 35.6%). The PPV was low (9.9% for M to 15.8%) while the NPV was high (79.2% to 93.8%). Use of reported LAP alone would have resulted in less than half of infected women getting treatment. In addition, the kappa score was very poor for all the STIs.

Combining the two commonly occurring symptoms (vaginal discharge and LAP) would have led to a reduction of overtreatment and an improvement in correct treatment rates. However, the kappa score remained poor (Table 3 (suite)). Combining three (discharge, LAP, dysuria) and four (discharge, LAP, dysuria, abnormal bleeding) symptoms generally had low sensitivity and PPV, high specificity and NPV and high missed treatment rates. The overtreatment rates were low and correct treatment rates were high. The kappa rates were very poor (Table 3 (suite)).

**Table 3(suite):** performance of reported symptoms

	Sens	Spec	PPV	NPV	Missed treatment	Over treatment	Correct treatment	k
**Vaginal Discharge and Pruritus**								
NG	46.2	44.7	15.0	79.7	53.8	55.3	45.0	-0.05
CT	61.1	47.3	13.8	89.9	38.9	52.7	49.0	0.03
TV	64.3	47.4	11.3	92.8	35.7	52.6	49.0	0.04
MG	33.3	45.0	3.8	91.3	66.7	55.0	44.3	-0.05
NG/CT	40.0	45.8	2.5	95.7	60.0	54.2	45.6	-0.02
**LAP syndrome (vaginal discharge, LAP, dysuria, abnormal bleeding)**								
LAP								
NG	61.5	30.9	15.8	79.2	38.5	69.1	36.2	-0.04
CT	61.1	31.3	10.9	85.4	38.9	68.7	34.9	-0.03
TV	71.4	35.6	9.9	91.7	28.6	64.4	35.6	0.01
MG	55.6	31.4	5.0	91.7	44.4	68.6	34.4	-0.02
NG/CT	40.0	31.3	1.98	93.8	60.0	68.8	31.5	-0.03
**Vaginal discharge and LAP**								
NG	46.2	39.0	13.8	77.4	53.8	61.0	40.3	-0.08
CT	55.6	41.2	11.5	87.1	44.4	58.8	43.0	-0.01
TV	64.3	42.2	10.3	91.9	35.7	57.8	44.3	0.02
MG	22.2	39.3	2.3	88.7	77.8	60.7	38.3	-0.08
NG/CT	40.0	41.0	2.3	95.2	60.0	59.0	40.9	-0.02
**Vaginal discharge and LAP and dysuria**								
NG	30.8	74.0	20.0	83.5	69.2	26.0	66.4	0.04
CT	27.8	73.3	12.5	88.1	72.2	26.7	67.8	0.01
TV	14.3	71.9	5.0	89.0	85.7	28.1	66.4	-0.08
MG	11.1	72.1	2.5	92.7	88.9	27.9	68.5	-0.06
NG/CT	20.0	73.0	2.5	96.3	80.0	27.0	71.1	-0.02
Vaginal discharge and LAP and dysuria and abnormal bleeding*								
NG	11.5	96.8	42.9	83.8	88.5	3.2	81.9	0.12
CT	0.0	94.7	0.0	87.3	100.0	5.3	83.2	-0.07
TV	7.1	95.6	14.3	90.8	92.9	4.4	87.3	0.03
MG	0.0	95.0	0.0	93.7	100.0	5.0	89.3	-0.06
NG/CT	0.0	95.1	0.0	96.5	100.0	4.9	92.0	-0.04

Sens: sensitivity; spec; specificity; PPV: positive predictive value; NPV: negative predictive value; LAP: lower abdominal pain; *abnormal bleeding = postcoital bleeding and intermenstrual bleeding

## Discussion

Our study showed poor diagnostic accuracy of the vaginal discharge syndrome for the diagnosis of cervical infections. However, it showed acceptable accuracy for the screening of vaginal infections by *Trichomonas vaginalis*. The lower abdominal pain syndrome, used to screen for PID, performed very poorly in the diagnosis of the major causes of PID: *Neisseria gonorrhoeae, Chlamydia trachomatis* and *Mycoplasma genitalium*. On the other hand, the urethral discharge syndrome in men had acceptable performance for the screening of the four STIs. Generally, however, there were high rates of overtreatment and only over half of patients with infection, even for the best performing STI, NG, would have been correctly diagnosed with the use of the syndromic approach alone. NG was the commonest diagnosed STI among both men and women aged above 25 years. For those under 25 years, CT was the commonest diagnosed STI in females while NG was the commonest among males. In addition, CT infection was more likely to be diagnosed in women aged less than 25 years (p=0.004).

The poor diagnostic accuracy of VDS for the diagnosis of cervical infections, has been previously documented in different populations and geographical regions [4,15-18]. That this approach has performed poorly in an STI clinic setup, where individuals are likely to have self-selected from perceived risk factors is worrying and calls for a rethinking of the diagnosis of cervical infections using the syndromic approach.

The urethral discharge syndrome for the diagnosis of STI in men is adequate has previously been shown [16,19]. Similarly, the performance of VDS for vaginal infections has been adequate in other studies [5]. Indeed, previous studies have called for VDS to be used exclusively for the diagnosis of vaginal infections [4]. However, the high overtreatment rates limit their usefulness because of subsequent risk of antimicrobial resistance from the use of broad-spectrum antimicrobial agents [9]. With the rise in antimicrobial resistance in NG and MG isolates becoming more prevalent [20], introduction of the aetiologic diagnosis into the UDS and VDS models should be considered. The fact that LAPS performed so poorly in the diagnosis of the potential causes of PID is of great concern. This is because, PID can lead to serious reproductive complications in women such as tubal factor infertility [21] and a screening test with poor accuracy will lead to undiagnosed and therefore untreated cases of PID.

Improvement of STI diagnosis will require a shift from syndromic to aetiologic diagnosis as proposed by the WHO [9]. This requires the availability of point-of-care tests (POCTs) and their integration into the healthcare system [22] especially in low- and middle-income countries (LMICs) which carry the highest burden of curable STIs [23]. Indeed, high diagnostic accuracy and acceptability of POCTs targeting NG, CT and MG in an LMIC setting with high HIV prevalence has already been demonstrated [24,25].

One major setback of the syndromic management is asymptomatic infection. Our study shows asymptomatic infections of between 6.8% for NG in men and 22.2% for MG in women. These findings are similar to others that have found asymptomatic infections being an impediment to the use of the syndromic approach [26,27]. Indeed, these individuals would have remained untreated without the aetiologic diagnosis. These and previous findings stress the importance of the eventual move from syndromic to aetiologic management of STIs.

Our findings that *Chlamydia trachomatis* was the commonest infection among young women (18-24 years) is consistent with other studies that have shown that this infection is commoner in adolescent and young women [26,28]. It is on this basis that CT screening is recommended for sexually active young women to prevent sequelae such as PID and infertility [29]. Unfortunately, this recommendation has not yet been implemented in Kenya. Similarly, NG infection is not routinely screened for among sexually active young men. This, coupled with the rising incidence of antimicrobial resistance means that concerted efforts are needed to stem this rise.

Our study was not without limitations. Firstly, we used reported symptoms to calculate the diagnostic accuracy. The Kenyan guidelines recommend physical examination of the patients in addition to using reported symptoms for diagnosis. However, the use of reported symptoms alone is likely a reflection of what happens in many public health facilities due to lack of adequate staff and equipment. Secondly, we did not screen for bacterial vaginosis (BV) and vulvovaginal candidiasis (VVC) which account for majority of cases of vaginal discharge [30]. This may have underestimated the diagnostic accuracy of vaginal discharge.

## Conclusion

Our study found poor diagnostic accuracy for the use vaginal discharge and lower abdominal pain syndromes for cervical infections caused by NG, CT and MG. In addition, despite the accuracy being adequate for screening for TV infection in women and NG, CT, MG and TV infections in men, the high overtreatment rates and the low rates of correct treatment are a cause for concern. There is an urgent need for the review of the syndromic approach to the diagnosis of STIs which should include the introduction of POCTs in public health facilities. Further, that the CT prevalence in this population is more likely in young women calls for the introduction of recommendations of screening of sexually active young women.

### What is known about this topic

- The diagnostic accuracy of the syndromic approach to treatment of STIs is poor for cervical infections;
- The approach also leads to high cases of missed and overtreatment.

### What this study adds

- This study adds to the knowledge of the diagnostic accuracy of the syndromic approach to screening and case finding of Mycoplasma genitalium, an emerging STI;
- It also adds to the voice calling for the adoption of the aetiologic approach to the management of STIs in Kenya due to the inaccuracy of the syndromic approach;
- In addition, it shows the importance of screening for Chlamydia trachomatis and Neisseria gonorrhoeae in sexually active young adults, a practice which has not been adopted in Kenya.
